# Symbiosis of Plants with Mycorrhizal and Endophytic Fungi

**DOI:** 10.3390/plants12081688

**Published:** 2023-04-18

**Authors:** Raul S. Lavado, Viviana M. Chiocchio

**Affiliations:** Facultad de Agronomía, Universidad de Buenos Aires and Instituto de Investigaciones en Biociencias Agrícolas y Ambientales—INBA (CONICET/UBA), Av. San Martín 4453, Buenos Aires 1417, Argentina

## 1. Introduction

It has long been known that plants and microorganisms coexist. Thus, in the roots of vascular plants, there are a great variety of fungi (arbuscular mycorrhizal fungi, dark septate endophytes fungi (DSE) and other endophytes) establishing different types of relationships. These fungi can grow in biotrophic, saprophytic and even parasitic ways and often overlap functionally and ecologically with other soil fungi, such as saprotrophs, facultative or obligate pathogens. Due to this great heterogeneity of behavior, they have many interactions with their hosts. To facilitate the understanding of such interactions, this topic can be analyzed from four main points of view:

1. Effect of fungi on plant root architecture;
2. Effect of fungi on the water and nutrient plant uptake;
3. Effect of fungi on plant growing in contaminated environments;
4. Effect of fungi on organisms causing plant diseases, insects and weeds, which negatively impacts plant growth.

Regarding the first point, we discuss changes in host root architecture caused by the production of precursors of plant hormones (cytokinins, auxins and strigolactones). This effect is magnified by the production of extraradical hyphae, which, in the case of mycorrhizal fungi, give rise to highly functional absorbing networks. Both effects allow the roots to explore greater soil volume and to access to small soil pores and microsites, which are inaccessible to roots [1,2,3]. Secondly, fungal root colonization contributes to the uptake of nutrients by plants. For such effects, fungi produce and secrete extracellular enzymes, organic acids (among them citric, oxalic, malic and gluconic acids), chelating substances, phytosiderophores, etc. They degrade or solubilize organic and inorganic soil components, sometimes recalcitrant sources, and release their components, including nutrients. This is, perhaps, the most recognized among the many functions of symbiotic fungi [4,5,6]. Regarding the third point, other fungi reduce the effect of different natural or man-made stresses on plants. As an example, they limit the plant absorption and tissue accumulation of heavy metals. Moreover, fungi not only tolerate but degrade hydrocarbons, agrochemicals and other organic pollutants, supported by their ability to produce biosurfactants [7,8]. As a consequence, fungi use the contaminants as a carbon source. Finally, it is also known that mycorrhizal fungi and certain endophytic fungi neutralize the negative effects produced by micro and macro pathogenic organisms [9,10,11].

**Figure 1:**
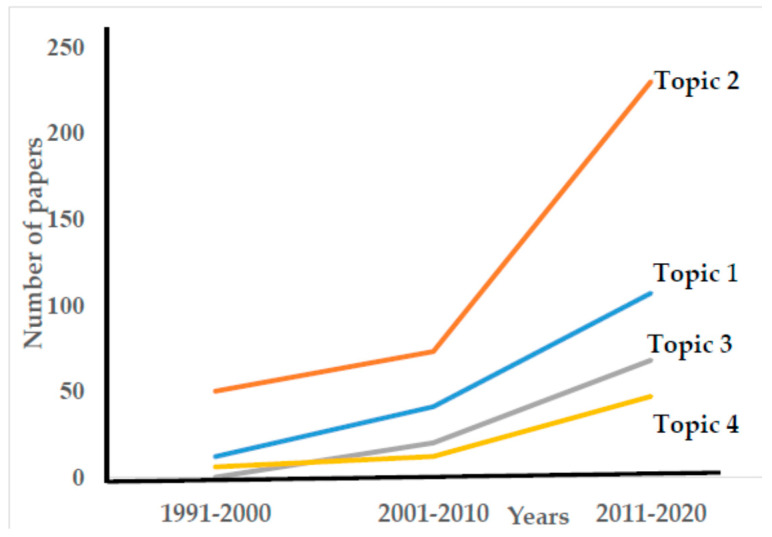
Evolution of the number of papers about mycorrhizal and endophytic fungi published, in the last three decades.

To quantify the importance of these four fields of symbiosis among fungi and superior plants, we utilized the “Scopus” Biological Sciences and Agriculture database and obtained the information shown in the following Figure 1:

In 30 years, publications on the fungus/root architectural relationship increased by almost nine-fold, the influence of fungi on nutrient and water uptake increased by almost five-fold, issues related to contamination increased by sixty-eight-fold (undoubtedly a recent topic) and the effect of fungi on harmful organisms increased by almost eight-fold. The influence of fungi on nutrient and water uptake is the topic that grew the least; however, more than 50% of the total papers were published in this field.

Relatively speaking, the average proportion of the topic “symbiosis among fungus and plant”, which constituted 0.08% of the large number of papers published in the biology and agronomy areas in the 1990s, grew to 0.17% in the following decade and reached the 0.54% in the 2010s. While our search was likely not comprehensive, it nevertheless demonstrates the growing importance of the present issue. The number of papers concerning plant–microorganism relationships increased by almost seven-fold in the last 30 years.

## 2. Special Issue Contents

This Special Issue covers the recent developments and future trends of this topic. It provides a perspective that will allow the reader to extend their understanding of the questions regarding the interactions that can be established between plants and microorganisms. Many papers included in this Special Issue studied the interactions of these endophytic fungi with different organisms (pathogenic fungi, insects and weeds) that have very negative effects on host plants. This is not a new topic, but the studies included here reveal that the knowledge of the interactions of these fungi with pathogenic or parasitic organisms has deepened in recent times, which is interesting when one considers that this topic was the least studied in previous years.

In this context, Yurkov et al. [12] focused on the study of the interaction of the plant and the early stages of the development of arbuscular mycorrhizae (AM), studying the metabolome of the inoculated root of *Medicago lupulina* in a substrate with low phosphorus. The results obtained indicated that mycorrhization was the determinant of the root metabolite profile rather than the further development of the host plant.

On the other hand, Koziol et al. [13] studied soil microbial communities and arbuscular mycorrhizal fungi and their effect on the establishment of native and non-native seeds. These authors concluded the importance of modifying soils with late-successional microbes, including native AM fungi, at the beginning of the restoration processes and that the dynamics of native and non-native seedlings can determine restoration results for many years.

In cultivated soils, Tosi et al. [14] studied the relationship between the phylogenetic composition of AMF, a cover crop of four species subjected to drought or irrigation. As a result of this research, it can be deduced that cover crops are able to affect the structure of AMF communities in the soil and modulate their response to levels of water availability. The heterogeneity of the soil could be an influential factor in the final result.

Nchu et al. [15] deepened the relationship between the endophyte fungus and the plant pathogen. They studied the effects of *Beauveria bassiana* on the growth of tomato plants infected with *Fusarium oxysporum f. *sp*. Lycopersici*. They concluded that *B. bassiana* only improved antioxidant capacity in healthy plants but not in those infected with *F. oxysporum*. An interesting result is that *B. bassiana* improved the plant growth of infected tomatoes and induced higher oxidative stress in *F. oxysporum* infected and non-infected tomatoes. Devi et al. [16] also worked with *Fusarium oxysporum*, but in this case, they evaluated the therapeutic potential of the medicinal plant *Ocimum tenuiflorum* through inoculation with endophytic fungi. They found a *Fusarium* strain, *Fusarium fujikuroi*, with strong antagonistic activity towards the fungal pathogens *Rosellinia necatrix* and *Fusarium oxysporum*. The authors emphasized the importance that this same effect can have on other medicinal plants by increasing their phytochemical content.

Three papers present an approach to AM fungi and their relationship with host growth. In the study of Conceição et al. [17] the role of AM as growth promoters of wheat plants and protection against abiotic stress was evaluated. It appears that both the parent plant type and soil disturbance affect wheat growth and the functional soil microbiome. Following this concept of the positive effects of AM fungi on plant growth and nutritional status, Duell et al. [18] evaluated the potential benefits and negative consequences of six commercial AM fungi inoculants on native and invasive plants. This paper discusses the possible ecological consequences of the inoculation of commercial products formulated with AM fungi. It concludes with the importance of evaluating local or native AM communities before applying AM fungi, as it may be unnecessary or possibly harmful. Starting from the premise that the success of the response of plants to AM inoculation depends on the plant–fungus combination, Säle et al. [19] with AM were negative or neutral on their growth. This work contributes to the theory that some weeds do not benefit from higher growth in the presence of AM and can even be negatively affected, which is an interesting outcome for organic farming.

The work of Pescie et al. [20] analyzed the effect of the inoculation of *Oidiodendron maius* A, *O. maius* BP, and *Acanthomyces lecanii* BC on the growth and survival of the seedlings of two southern high blueberry cultivars. It was found that *O. maius* BP had a positive effect on the N content and the plant biomass of both blueberry varieties, while *O. maius* A improved plant survival. These results show the importance not only of the species but also of the different strains, which may have complementary effects among themselves.

In another line of research, Chitti and Gange [21] presented the effects of insect herbivory and the fungal colonization of AM over generations of an annual herb, *Senecio vulgaris*. In the second generation, plant parameters were negatively affected, but after a few generations, these parameters reverted to similar values as those of the original generation. This shows the importance of monitoring several generations of plants when studying maternal effects, also taking into account biotic and abiotic factors.

Finally, this Special Issue is completed by the work of Bensaci et al. [22], who evaluated the effect of formulations from endophytic fungal filtrates on potatoes against the biotic stresses produced by *Myzus persicae* Sulzer. The application of these formulations significantly reduced the development of the early maturity of the embryos, resulting in a real possibility for the effective control of the insect in potato crops in an environmentally sustainable way.

Thus, the increase in our knowledge about the functions of plant–endophytic fungi relationships expands the potential application of microbe-based applications in agricultural and environmental management.

This Special Issue contributes by proposing solutions for more sustainable agrosystems, based on natural processes, in order to overcome different environmental problems that currently exist in our society.
